# Supplementary material for: Double Network Physical Crosslinked Hydrogel for Healing Skin Wounds: New Formulation Based on Polysaccharides and Zn2+
Source: Int J Mol Sci. 2023 Aug 22;24(17):13042. doi: 10.3390/ijms241713042 (PMC10488206; doi:10.3390/ijms241713042)
Supplement: Supplementary file 1 [file ijms-24-13042-s001.zip › ijms-2535932-supplementary.pdf]

Double Network Physical Crosslinked Hydrogel for Healing Skin Wounds: New Formulation Based on Polysaccharides and Zn^{2+}

Shenghao Cui, Faming Yang, Dingyi Yu, Chao Shi, Di Zhao, Liqi Chen, and Jingdi Chen *

Marine College, Shandong University, Weihai 264209, 202000810226@mail.sdu.edu.cn (S.C.); 202121023@mail.sdu.edu.cn (F.Y.); 202000810156@mail.sdu.edu.cn (D.Y.); 201936673@mail.sdu.edu.cn (C.S.); 201900810068@mail.sdu.edu.cn (D.Z.); 201900810011@mail.sdu.edu.cn (L.C.)

Corresponding Author

* Jingdi Chen, Ph.D.

Marine College, Shandong University, No. 180, Wenhuaxi Road, Weihai 264209 China

Tel: +86 6315688303

E-mail addresses: jdchen@sdu.edu.cn (J.C.)

Supplementary Methods

Preparation method of SA/CS/Zn^{2+} PDH

Different amounts of basic zinc carbonate (0, 10, 20 and 30 mM Zn^{2+}) add to 1.0wt% SA solution, then sonicated for 30 min and stirred for 60 min. 2.0 wt% of the total polymer was prepared by dispersing 1 g of CS powder into the SA solution containing Zn^{2+}, and sonicated for 60 min and stirred overnight. When the CS powder was completely dispersed in the SA solution, 0.5 g GDL was added. All the SA/CS/Zn^{2+}PDH were formed within 2 h. The SA/CS/Zn^{2+}PDH were recorded as SA-

CS-0, SA-CS-10, SA-CS-20 and SA-CS-30 due to the different Zn^{2+} content in SA/CS/Zn^{2+}PDH.

Gelation time

The vial tilt method was used to estimate the time of gel formation. A certain amount of GDL was added to the samples with different zinc contents; the addition process was continuously stirred to mix well until the liquid was no longer flowing, and the gelation time was recorded. Each group of experiments was repeated three times and the mean was taken.

Physico-chemical properties of SA/CS/Zn^{2+} PDH

Electron scanning microscopy (SEM) was used for observation. The completely dried hydrogel sample particles were powdered and fixed on double-sided conductive adhesive, and the samples were sprayed with gold under vacuum, while the voltage was adjusted to 12.5 KV.

FTIR was performed using an IRSpirit-1 instrument (Shimadzu, Japan). The hydrogels were dried and ground into powder, and the powder samples were mixed well with KBr. The hydrogel spectrum ranged from 4000 to 500 cm^{-1}, and a total of 32 scans were performed with a resolution of 4 cm^{-1}. Background measurements were performed using potassium bromide thin slices.

A thermal analyzer (TGA 449C, TA Instruments-Waters LLC, USA) was used to evaluate the thermal stability performance of this double network hydrogel. The samples after freeze-drying were heated from 30 °C to 800 °C in an atmosphere with a nitrogen flow rate of 50 mL/min at a heating rate of 10 °C/min.

Swelling property

The swelling behavior of the hydrogel was evaluated in PBS buffer. The pre-weighed freeze-dried samples were soaked in PBS for one day, and the samples were taken out every two hours and the excess water was absorbed by filter paper and then weighed. The swelling ratio (Q) of the hydrogel were calculated according to the following equations, respectively:

$$Q(\%) = (Wt - W0)/W0 \times 100\%$$

Where W_t and W_0 are the weight of the expanded hydrogel and the weight of the lyophilized hydrogel, respectively.

Water Retention Capacity

The pre-weighted swollen hydrogel sample (W_{eq}) equilibrated in distilled water was placed in a vacuum oven at 80 °C. The swollen hydrogel was weighed every 2 h (W_t), and then the percentage water retention (R) was calculated using the Eq.

$$R(\%) = (Wt - Wd)/(Weq - Wd) \times 100\%$$

Where W_d is the freeze-dried weight of hydrogel sample before swelling.

Mechanical performance testing

The standard number of the mechanical test is GB / T 16491-2008. The texture profile analysis (TPA) of the hydrogel was performed using the XT Plus Texture Analyzer (Stable Micro Systems, UK) to evaluate the spring, gumminess, cohesiveness, hardness, adhesiveness, and cohesiveness of the hydrogel. All measurements were made on gels equilibrated to room temperature. The gel samples were placed between parallel flat plate fixtures. The gels were compressed twice at 70% deformation and

with a relaxation time of 30s.

The mechanical properties of the hydrogels were evaluated by uniaxial compression experiments using a universal test machine (HounsefieldH25K, UK). Mechanical tensile tests were performed using constant crosshead speed of 1 mm/min, dumbbell shaped samples cut out from hydrogels and characterized with concave width of 10 mm and thickness of 2 mm. The gauge was a = 5 mm and sampling frequency was 10 Hz.

$$\lambda = \frac{a+b}{a}$$

In the formula: 'λ' corresponds to deformation of sample, while 'a' represents the standard length of materials, 'b' represents the mixture moving distance.

Frequency scan experiments were performed using AntonPa1-100 rad with a strain of 1%, using a MCR101 rheometer (Anton Paar, AT) with a parallel plate (Ø50 mm). The viscoelastic modulus of the samples was examined by measuring the energy storage modulus and the loss modulus. The optimal strain was predetermined from strain scan experiments to ensure that measurements were made within the linear viscoelastic strain range. Wet hydrogel samples were made into discs and analyzed using a parallel plate geometry with a diameter of 20 mm while adjusting the gap between the plates to 1 mm.

In Vitro **Release of Zn^{2+}**

The content of Zn^{2+} in the hydrogel was determined by inductively coupled plasma mass spectrometry (ICP-MS) (Thermo Scientific, XSERIES 2, USA). The test hydrogels (cylindrical, diameter 15mm, thickness 6mm) were placed in a four-well

plate with 10ml PBS buffer (PH = 7.4) in each well. After that, they were placed in an incubator at 37 °C and placed for one day at 75 % relative humidity. PBS solutions were collected at the specified time (1,2,4,6,12,24h), and all samples were measured by ICP-MS. The release rate of Zn^{2+} is calculated by the following formula:

$$Release\ rate(\%) = (C0 * VPBS)/(MZn * n0) \times 100\%$$

In the formula: C_0 is the measured data (unit, μg / L) ; V_{PBS} was the volume of PBS used (V_{PBS} = 10ml) ; M_{Zn} is the molar mass of zinc ; n_0 is the total molar amount of zinc in the sample (n0 = 0.005 * 0.01、0.02 or 0.03 mol).

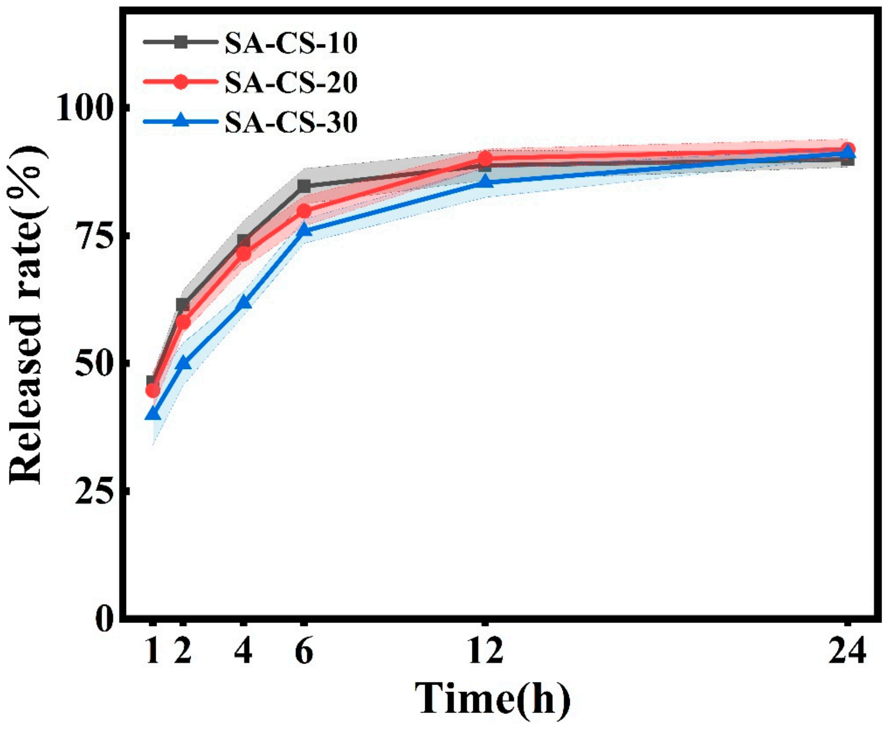

Figure S1: the Zn^{2+} release rate of hydrogel(n=3).

Antioxidant activity of SA/CS/Zn2+ PDH *in vitro*

The freeze-dried hydrogel of the same quality was accurately weighed and mixed with 2ml PBS buffer to form a 10mg / ml solution. 2ml material solution was mixed

with 2ml DPPH (150μmol / L) solution and reacted in the dark for 30 min. The sample was placed in a microplate reader, and the absorbance at the wavelength of 517 nm was measured after shaking for 60 s. Ascorbic acid (5%) as a positive control. DPPH free radical scavenging rate calculation formula:

$$Clearance\ rate(\%) = (Ai - Aj)/Ai \times 100\%$$

In the formula: A_i is the blank absorbance; A_j is the absorbance of the sample.

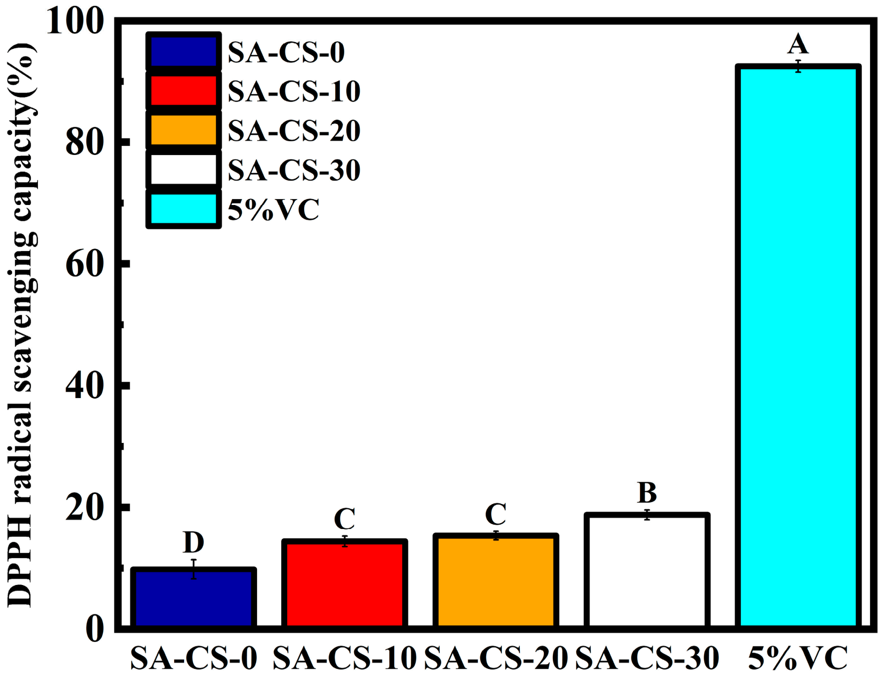

Figure S2: DPPH free radical scavenging rate of hydrogel (n=3).

In vitro evaluation of antibacterial properties

The inhibition activity of the hydrogels against two typical Gram-negative and positive bacteria, *E. coli* and *S. aureus,* were evaluated using the disc diffusion method. The diameter of each hydrogel for the disc diffusion test was 15 mm. The hydrogel samples were cut into small discs of the same size, sterilized under UV lamp

for 30 min, and all the experimental supplies were transferred to a sterile operating table and marked well; 15 mL of solid culture was measured based on the surface dish, 100 μL of the cultured bacterial solution was taken, and the surface dish was shaken to mix the bacterial solution evenly, and left to stand for 20 min before lamination. The plate was transferred to a constant temperature incubator and incubated at 37 °C for 24 h. The experimental results were observed.

Biocompatibility testing

The cytotoxicity of SA/CS/Zn^{2+}PDH was assessed by the CCK-8 method. HaCaT cell solution (100 μL, approximately 2 × 104 cells/mL) was inoculated into 96-well plates. After incubation at 37 °C for 24 h, the supernatant was discarded. The extracts of the samples (CS-SA-0, CS-SA-10, CS-SA-20, CS-SA-30) and positive control) were sterilized under ultraviolet lamp for 2h. Then, sample solutions configured from fresh medium (HaCaT DMEM) were added to each well and incubated for 24 h. CCK-8 reagent (10 μL) was added and incubated for 4 h. A microplate reader (Epoch2NS, BioTek, USA) was used to measure the absorbance at 450 nm. All experiments were performed in quintuplicate.

Cell viability was respectively determined by AO/EB double fluorescence staining method. Each sample was irradiated under ultraviolet lamp for 2h. AO/EB working solution was added to each cell sample and mixed well. Centrifuge at low speed for 5 min and discard the supernatant. Cells were resuspended with AO/EB Dilution Buffer and counted, and the cell density was adjusted to 0.5-5×10^6 cells/mL. For every 25 μL of cell suspension, AO/EB working solution was added and mixed thoroughly for

observation in the fluorescence microscope.

***In vivo* wound healing test**

Construction of a wound repair model

All animal experiments were performed in accordance with the experimental protocols reviewed and approved by the Shandong University Laboratory Animal Center, and in strict compliance with the Institutional Animal Care and Use Committee (IACUC) guidelines for the ethical use of animal models in biological research. Mice were randomly divided into 7 groups of 9 mice each. A composite anesthetic was injected intraperitoneally into them with shaved dorsum, and a 0.8 cm diameter all-cortical trauma model was created on the dorsum of each mouse. Groups 1-5 corresponded to Zn^{2+} levels of 0, 5, 10, 20, and 30 mM/L, group 6 was a blank group (no treatment), and group 7 was a control group coated with petroleum jelly. The precursor solutions of hydrogel and saline were sterilized by filtration using a 200 nm syringe-type filter. After surgery, the mice were kept alone in cages to allow them to heal, after which the wounds were photographed every other day with a camera (EOS850D, Canon, Japan). Wound healing rates were estimated using Image J (National Institutes of Health, Bethesda, MD, USA). Wound healing rates were calculated based on the following:

$$\text{Wound healing (\%)} = \frac{D_0 - D_n}{D_0} \times 100\%$$

Where D_0 is the diameter of the wound at the time of modeling, D_n is the diameter of the wound at the specified point in time.

Histological analysis

3, 7, and 14 days after surgery, trauma sections were taken, fixed with 4% paraformaldehyde, and paraffin-embedded sections were used for subsequent preparation of sections for H&E and Masson staining.

The sections were transferred to dye jars containing dd H_2O, soaked and washed for 3-5 min; stained with hematoxylin solution for 20 min and washed with water; color separation with 0.5% hydrochloric acid for 3-10 s and rinsed in running tap water for >30 min until blue color was observed; samples were respectively treated with 50%, 70% and 80% ethanol for 5 min and stained with eosin (diluted by 95% ethanol solution) for 1 min. The samples were washed twice with 95% ethanol and twice with anhydrous ethanol for 5 min/time; washed 3 times with xylene solution for 5 min/time for complete dehydration; sealed with neutral gum and dried in a fume hood. Finally, the H&E staining results were observed using an orthomosaic microscope.

Slides were stained in a staining jar and deparaffinized by immersing them in two series of absolute xylene for 2 min each, followed by two rehydration processes with absolute ethanol, 90 % and 80 % for 2 min each. The slides were then immersed in Bouin's solution for 45 min at 60° Celsius. The slides were then cleaned in flowing tap water until the yellow color was removed. hematoxylin was applied to the slides for 8 min before being rinsed in running tap water for 2 min. Slides were immersed in acid fuchsin for 5 min before being rinsed with running tap water for 2 min. After that, the slides were treated for 10 min in phosphomolybdic acid solution and 5 min in methyl blue, respectively. The slides were then cleaned in running tap water for 2 min before being immersed in 1 % acetic acid for 1 min. The slides were dehydrated in 80 %, 90 %,

and 100 % ethanol before being immersed in absolute xylene for 1 min at each phase.

Finally, the Masson staining results were observed using a light microscope.
